# Child maltreatment among deaf and hard-of-hearing adolescent students: associations with depression and anxiety

**DOI:** 10.3389/fpsyg.2024.1287741

**Published:** 2024-02-13

**Authors:** Mohammad Ahmed Hammad, Mansour Nayef Al-Otaibi, Huda Shaaban Awed

**Affiliations:** ^1^Department of Special Education, College of Education, Najran University, Najran, Saudi Arabia; ^2^Department of Education and Psychology, College of Education, Najran University, Najran, Saudi Arabia

**Keywords:** child maltreatment, deaf and hard-of-hearing, depression, anxiety, Saudi Arabia

## Abstract

**Objective:**

Child abuse and neglect have several short- and long-term consequences for the victim. Though Deaf and Hard-of-Hearing children are at higher risk of being maltreated as compared to hearing children, little research in Saudi Arabia has focused on this population. To determine the prevalence of child maltreatment and to examine its association with depression and anxiety among a sample of Deaf and Hard-of-Hearing students in Saudi Arabia, recruited from secondary schools in southern Saudi Arabia.

**Methods:**

The sample included 186 Deaf and Hard-of-Hearing students aged 14–17 years (*M* = 15.7 years; SD = 3.41 years). Data were collected using the Child Abuse Self-Report Scale, Center for Epidemiological Studies Depression Scale for Children, and Generalized Anxiety Disorder Questionnaire. Bivariate and Linear regression analyses were conducted using SPSS 20.

**Results:**

About 47.3% of the students were exposed severe to very severe child maltreatment. The severity of maltreatment varied based on parents’ educational and income level, number of children in the family, the Deaf and Hard-of-Hearing student’s gender, and parents’ hearing status. Linear regression analysis indicated that child maltreatment was a significant predictor of depression and anxiety in this sample.

**Conclusion:**

Considering the socio-demographic factors influencing the prevalence of maltreatment in the present study, it seems important to work with parents of Deaf and Hard-of-Hearing children to improve their skills in rearing a child with special needs. Addressing the social stigma and social barriers experienced by DHH individuals through familial, institutional, and community interventions may be a first step toward long-term prevention of maltreatment among DHH children.

## 1 Introduction

Child maltreatment, including child abuse and neglect, lead to physical and psychological harm, and cause unwanted short- and long-term trauma to the victims. According to the World Health Organization (2022), child maltreatment includes “all forms of physical and emotional ill-treatment, sexual abuse, neglect, and exploitation that results in actual or potential harm to the child’s health, development or dignity.” Defined to include physical, sexual, and emotional abuse, and neglect (Hillis et al., 2016), child maltreatment continues to be highly prevalent despite substantial evidence of its negative effects. For instance, In the United States alone, there were about 3.5 million reported cases of child abuse each year (Health and Services, 2013). Another meta-analysis reported that the global number of abuse victims aged 2–17 years was 1 billion (Hillis et al., 2016). Children in low- and middle-income countries have been found to experience higher incidences of abuse, whether in the form of corporal punishment or psychological abuse (McCoy et al., 2020). In fact, as compared to Africa, Europe, Latin America and North America, Asia was found to have the highest incidence of abuse among children aged 2–14 years (68%) (Lansford et al., 2020). Parents are the most common perpetrators of physical and psychological abuse of their children (McCoy et al., 2020), with parents being the abuser in 61.4% of the reported cases (MacNiven et al., 2020). Nevertheless, non-parental abusers are also common, including but not limited to a relative, school personnel, family friend, another child, etc. (Abuse, 2014).

Children with disabilities, including those who are Deaf and Hard-of-Hearing (DHH) are more likely to be exposed to violence both at home and in the school environment than are their normal peers, and they may experience domestic violence at an earlier age than their normal peers (Turner et al., 2011; Koivula et al., 2018; Wakeland et al., 2018). Factors contributing to higher maltreatment vulnerability among persons with disabilities include increased physical, emotional, and financial dependence on others to meet their basic needs, poor ability to control their lives, and poor knowledge about sex and touch (e.g., “Good” vs. “Bad” touch) (Plummer and Findley, 2012). Furthermore, the communication difficulties experienced by DHH individuals may contribute to this high risk of being abused (Fellinger et al., 2012). Among DHH children, those born to hearing parents may experience substantial communication difficulties owing to a lack of family members’ knowledge of ways to communicate with their DHH child (Wakeland et al., 2018) or lack of social programs that support their learning of alternative communication methods such as sign language (Schwenke, 2019). Furthermore, lack of acceptance and the stigma of having a DHH child may cause negative familial attitudes, which may expose the child to aggression and abuse (Wakeland et al., 2018). Thus, poor communication may hinder the ability of DHH children to report abuse or identify sources of assistance within the community (Fellinger et al., 2012). Further, the stigma, social isolation, and rejection associated with hearing disabilities may contribute to the higher vulnerability to abuse. For instance, DHH individuals may sometimes be viewed as inferior or less humane than their hearing peers, and therefore, they may be more vulnerable to aggression and abuse as compared to their hearing peers (Admire and Ramirez, 2021).

Landsberger et al. (2014) reported that more than a third of young DHH individuals experience poor family relationships, suicidal behavior, poor communication, poor mental and physical health, and a higher incidence of certain clinical disorders. As compared to hearing peers, DHH children are more vulnerable to emotional abuse and neglect (Admire and Ramirez, 2021) physical abuse (Titus, 2010), and sexual abuse (Francavillo, 2009), with the incidence of sexual abuse being as high as 50% among children who experience communication difficulties (Francavillo, 2009). The high rate of sexual assault among DHH children may be because they are perceived as less likely to report the abuse, may not be aware that the sexual assault behavior is illegal, or may have limited sexual awareness (Denmark, 1994; Wakeland et al., 2018).

A large body of research has confirmed that child abuse and neglect are closely associated with the emergence of various mental illnesses (Norman et al., 2012), including but not limited to depression (Nelson et al., 2017) and anxiety disorders (Hovens et al., 2015). However, the incidence of mental health problems among DHH children is substantially higher than that among their hearing peers (Lightfoot and Williams, 2009; Fellinger et al., 2012). Nevertheless, being a population minority, there is little research on the mental health outcomes of DHH individuals (Barnett et al., 2011; Landsberger et al., 2014). This is also true in the Saudi context. Though not conclusive, extant literature suggests a 50–75% prevalence rate for child abuse in Saudi Arabia (Mogaddam et al., 2015; Almuneef M. et al., 2016). Almuneef M. A. et al. (2016) also reported that childhood abuse and violence were linked to increased rates of anxiety, depression, mental illness, drug use, smoking and alcohol dependence. However, the present authors failed to find similar studies conducted on the DHH population.

Though studies from other contexts show that *D*HH children are more vulnerable to child abuse and neglect, the prevalence of such maltreatments among Saudi DHH children, and their its effects on their mental health are currently unknown. Accordingly, the present study aimed to fill a research gap in the Saudi context, by exploring the following research questions:

1. What is the prevalence and severity of child abuse and neglect among the sample of DHH students in Saudi Arabia?
2. Does child abuse and neglect predict the level of depression and anxiety among the present sample of DHH students in Saudi Arabia?

## 2 Methodology

### 2.1 Participants

The current sample consisted of 186 DHH students enrolled in integration programs offered in southern Saudi Arabia (age range = 14–17 years; *M* = 15.7 years, SD = 3.41 years). Using G*Power version 3.1.9.7, we computed the recommended sample size for all the statistical tests used, with 0.05 α error probability and 85% power. The highest recommended sample size was for One-way ANOVA, which was 180. Thus, our final sample met the requirement. Purposive sampling was used to select participants who fit the following selection criteria: being aged 14–17 years, being identified as DHH (with hearing impairment level ranging from severe to profound hearing loss (> 81 dB HL), as recorded in the school reports), absence of other disabilities (confirmed through school reports), being able to communicate in sign language, which was the primary mode of communication used by the present researchers, providing voluntary informed assent to participate in the study, and who received voluntary informed consent from parents. Table 1 presents the detailed demographic information of the DHH participants.

**Table 1:** Demographic characteristics of the study participants.

Demographic characteristics		*f*	%
Participants’ gender	Men	102	54.83
	Women	84	45.61
Parents’ educational level	High school or less	98	21.31
	Graduation	66	40.65
	Higher than graduation	22	38.03
Parents’ socio-economic level	High Level	89	29.18
	Middle Level	65	34.94
	Low Level	32	17.20
Number of children in the family	≤1 children	94	50.53
	2–4 children	59	31.72
	≥ 5 children	33	17.74
Parents’ hearing status	Hearing	139	74.73
	DHH	37	19.89

### 2.2 Measures

#### 2.2.1 Child abuse self-report scale

The Child Abuse Self-Report Scale (CASRS) (Kent and Waller, 1998) was developed to assess abuse and neglect experienced in the home environment. This self-report, 38-item questionnaire assess children’s experience of four types of childhood maltreatment, namely, physical abuse (8 items) sexual abuse (5 items), emotional abuse (14 items), and neglect (11 items), which are rated on a 4-point Likert scale assessing the frequency of abuse. Total scores range from 0 to 152 points. Several studies have confirmed the reliability and validity of the CASRS (Kent and Waller, 1998; Mohammadkhani et al., 2003; Hadianfard, 2014). The current study confirmed good internal consistency in the DHH sample (Cronbach’s *α* = 0.87).

#### 2.2.2 Center for epidemiological studies depression scale for children

The Center for Epidemiological Studies Depression Scale for Children (CES-DC; Weissman et al., 1980) is a self-report tool to assess the severity of depressive symptoms in individuals aged 6 to 17 years. The scale comprises 20 items rated on a 4-point Likert scale, with scores ranging from 0 to 60. Items 4, 8, 12, and 16 are reverse scored, and a total score of over 15 points is considered to indicate depression children and adolescents. The CES-DC has exhibited good internal consistency reliability in several studies with normal hearing children and adolescents (Weissman et al., 1980; Barkmann et al., 2008; Betancourt et al., 2012), and with children with disabilities (Quintero and McIntyre, 2010; Pepa, 2013; Çelik et al., 2018). It exhibited good internal consistency in the present DHH sample (Cronbach’s *α* = 0.83).

#### 2.2.3 Generalized anxiety disorder questionnaire

The 7-item Generalized Anxiety Disorder Questionnaire (GAD-7; Spitzer et al., 2006) is a self-report tool that assesses the severity of generalized anxiety disorder symptoms based on DSM IV criteria. Assessing the frequency of symptoms experienced over the past 2 weeks, the GAD-7 takes approximately one to 2 min to administer. Each item is rated on a 3-point Likert scale, with total scores ranging from 0 to 21. Scores of 5, 10, and 15, are considered as cut-off points to indicate mild, moderate and severe anxiety symptoms, respectively. The scale showed good internal consistency (Cronbach *α* = 0.92) and reliability (correlation within category = 0.83) in the original study (Spitzer et al., 2006), as well as subsequent studies with children and teenagers with normal hearing (e.g., Mossman et al., 2017; Sun et al., 2021) and with children and adolescents with disabilities (Pozzatti, 2020; Hammad, 2023). In the current study, it showed good internal consistency in the DHH sample (Cronbach *α* = 0.77).

### 2.3 Data collection

For use in the present study, all three tools were translated from English to Arabic and then translated back into English by language experts, to ensure that the Arabic translation had the same meaning as the original questionnaire. First, the English version was translated into Arabic by a bilingual professor from the English Department in the researcher’s institution. The Arabic version was retranslated into English by another professor who specializes in English and whose first language is Arabic. Subsequently, the Arabic and English translated versions were reviewed by three experts specializing in Arabic, Psychology, and English, respectively. Based on the consensus among the three specialists, some words and clauses were revised to create the final Arabic version of the tools. As the present sample of students preferred to communicate in sign language, the final Arabic version was translated into sign language by a specialist who teaches DHH students. Three teachers who worked in the schools with the DHH students were assigned to communicate directly with the participants and administer the questionnaires. This was done because the teachers were fluent in sign language and had a trusting relationship with the DHH participants. Prior to data collection, the researchers explained key jargon to the teachers, such as child abuse, depression, generalized anxiety, etc., to help them communicate their meanings effectively to DHH students. Subsequently, the teachers were asked to read the questionnaires carefully, and the researchers and teachers came to an agreement on how to translate them into sign language. Finally, the tools were pilot tested with a 4 DHH students (who were not included in the study sample). They were asked to clarify difficult words and unclear elements, and suggest easier alternatives. After incorporating their feedback, the tools were administered to the study sample.

Data were collected from February to April 2023. Before collecting the data, all DHH student participants and their parents completed informed consent forms that explained that their participation was voluntary, that all information obtained would remain strictly confidential, and that the data would only be used for research purposes. Their right to decline participation at any point in the study was also explained. Additionally, before the study began, the researchers obtained ethics approval from the Deanship of Scientific Research at ABC University (No. NU/DRP/SEHRC/12/3). The data collection process took 75 min for each participant, after which, the teachers and students received a small gift in appreciation of their participation.

### 2.4 Data analysis

All statistical analyses were performed using SPSS version 20. The internal consistency of the three tools used in the study was assessed by computing Cronbach’s α coefficients because these tools had never been used in a Saudi sample of DHH students. Appropriate descriptive statistics (frequency and percentage or mean and standard deviation) were computed to examine the prevalence of child maltreatment and its severity in the present sample. Bivariate analyses (t-tests and ANOVA) were used to conduct comparisons across demographic variables. Finally, linear regression analysis was used to examine the relationship between child maltreatment and the two mental health variables of depression and anxiety scores. As explained later, appropriate control variables were included in the regression model. Statistical tests were calculated using guidelines from Cohen (Cohen, 2013).

## 3 Results

### 3.1 Child maltreatment among DHH students

Table 2 shows the prevalence of child abuse and neglect among the present sample of DHH students, across the 4 severity categories created by the researchers for the present analysis. Given that the CASRS does not provide specific cut-off points to determine the severity of maltreatment, the present authors utilized the following arbitrary classification for further analysis: 0–38 points: mild or no child maltreatment; 39–76 points: moderate maltreatment; 77–114 points: severe maltreatment; and ≥ 115 points: very severe maltreatment. Note that the psychometric properties of this classification have not been determined, and it has been used only for analytical purposes in the present study.

**Table 2:** Prevalence of child maltreatment among DHH students.

	No child maltreatment or mild *N* (%)	Moderate *N* (%)	Severe *N* (%)	Very severe *N* (%)	*M* (SD)
Emotional abuse	2 (1.07)	102 (54.83%)	68 (36.55)	14 (7.52)	2.51 (0.63)
Physical abuse	20 (10.75)	51 (27.41)	92 (49.46)	23 (12.63)	2.63 (0.83)
Neglect	3 (1.61)	54 (29.03)	85 (45.69)	44 (23.65)	2.96 (0.74)
Sexual abuse	93 (50.0)	88 (47.31)	5 (2.68)	0 (0)	1.52 (0.55)
CASRS Total	4 (2.68)	94 (50.53)	73 (39.24)	15 (8.06)	2.55 (0.64)

CASRS, Child Abuse Self-Report Scale.

The results showed that, of the 186 DHH participants, 92 (50%) were exposed to a moderate level of child maltreatment, and 88 (about 47.3%) were exposed to a level ranging from severe to very severe. All students experienced each type of abuse/neglect at least to some extent. Furthermore, nearly all DHH participants (184, about 99%) reported having experienced moderate to very severe levels of emotional abuse and neglect, 164 (89%) reported having experienced moderate to very severe levels of physical abuse, and 93 (50%) reported having experienced moderate to very severe levels of sexual abuse.

Subsequently, one-way ANOVAs were conducted to examine differences in CASRS scores based on demographic characteristics with more than 2 categories. Significant group differences in CASRS scores were observed for parents’ educational level (*F* = 13.496, *p* = 0.000), parents’ income level (*F* = 7.847, *p* = 0.001), and number of children in the family (*F* = 4.260, *p* = 0.016). To further assess the nature of these differences, Scheffe’s test was used for post-hoc analyses based on parents’ educational level, parents’ income level, and number of children in the family (Table 3).

**Table 3:** Post-hoc analyses results comparing CASRS scores based on demographic characteristics.

Variables		Mean difference* (I-J)	Std. Error	Sig.	95% CI	
					Lower bound	Upper bound
(I) Parents’ educational level	(J) Parents’ educational level					
Higher than graduation	Graduation	−19.63	0.000	1.678	−15.48	−11.32
	High school or less	−44.21	0.000	1.737	−39.91	35.61
Graduation	Higher than graduation	11.32	0.000	1.378	15.48	19.63
	High school or less	−28.45	0.000	1.623	−24.43	−20.41
High school or less	Higher than graduation	35.61	0.000	1.737	39.91	44.21
	Graduation	20.41	0.000	1.623	24.43	28.45
(I) Parents’ socio-economic level	(J) Parents’ socio-economic level					
High level	Medium Level	−20.59	0.000	1.64	−16.51	−12.44
	Low level	−43.78	0.000	1.78	−39.35	−34.92
Medium Level	High level	12.44	0.000	1.64	16.51	20.59
	Low level	−26.91	0.000	1.64	−22.83	−18.75
Low level	High level	34.92	0.000	1.78	39.35	43.78
	Medium Level	18.75	0.000	1.64	22.83	26.91
(I) Number of children in the family	(J) Number of children in the family					
1–2 children	3–4 children	−23.62	0.000	1.66	−19.50	−15.38
	≥ 5 children	−42.08	0.000	1.83	−37.54	−33.00
3–4 children	1–2 children	−15.83	0.000	1.66	19.50	23.62
	≥ 5 children	−22.08	0.000	1.63	−18.03	−13.98
≥ 5 children	1–2 children	33.00	0.000	1.83	37.54	42.08
	3–4 children	13.98	0.000	1.63	18.03	22.08

CASRS: Child Abuse Self-Report Scale. *All mean differences are significant at the 0.05 level.

As evident from Table 3, DHH students whose parents had a High school or less exhibited highest CASRS scores, followed by those whose parents had a graduation and Higher than graduation, respectively. Similarly, DHH students whose parents had a low income level exhibited highest CASRS scores, followed by those whose parents had medium and high income level, respectively. Finally, DHH students from families with ≥5 children exhibited highest CASRS scores, followed by those from families with 3–4 and 1–2 children, respectively. These results suggest that DHH children are more likely to experience abuse and neglect in families with more number of children, and when their parents have low educational and income levels.

For demographic variables with 2 comparison groups, a t-test was performed. Findings revealed that men participants tended to have higher CASRS scores as compared to their women counterparts (*t* = 9.58, *p* < 0.05; men s: *M* = 90.42, SD = 19.18; women s: *M* = 82.65, SD = 15.25). With reference to parents’ hearing status, participants with DHH parents had lower CASRS as compared to their peers with hearing parents (*t* = 9.16, *p* < 0.05; participants with DHH parents: *M* = 89.29, SD = 17.06; participants with hearing parents: *M* = 93.85, SD = 18.64).

### 3.2 Depression and anxiety among DHH students experiencing child maltreatment

As mentioned before, all students in the present sample experienced at least some form of aubse or neglect. Concurrently, the overall depression and anxiety scores in this sample were high. Specifically, out of a total possible score of 0–60 on the depression tool, the mean score in this sample was 15.38 (SD = 4.35). Using the recommended cut-off of 15 points to diagnose depression, it was found that 52.68% of the DHH students in the present sample fell in the “depressed” category. With reference to anxiety, out of a total possible score of 0–21 points, the mean score in this sample was 11.16 (SD = 2.75). Using the recommended cut-off of 5, 10, and 15 for mild, moderate, and high anxiety, it was found that majority (46.23%) of the DHH students in the present sample fell in the moderate, followed by those in the mild (21.03%) and high anxiety (19.82%) categories, respectively. Together, these findings confirm the high incidence of depression and anxiety among DHH students.

Table 4 presents the results of the linear regression analysis conducted to examine the relationship between DHH students’ maltreatment and mental health scores. Since all five demographic variables exhibited significant group differences in the CASRS scores, they were included as control variables in the analysis.

**Table 4:** Linear regression analysis of association between child maltreatment, and depression and anxiety.

Variables	B	Std. Error	Beta	*t*	*p*
Constant	6.38	62.21	9.74		0.00
Depression	0.397	1.67	4.20	0.339	0.00
Anxiety	0.58	1.82	4.20	0.23	0.002

*R* = 0.903; *R*^2^ = 0.815; Adjusted *R*^2^ = 0.807; *F* = 111.79, *p* < 0.05.

Gender, parents’ educational level, parents’ income level, number of children in the family, and parents’ hearing status were included as control variables.

The results revealed that depression and anxiety were significant predictors of maltreatment scores in the present sample of DHH students, with both variables together explaining over 80% of the variance in maltreatment scores. With every 1 point increase in the depression and anxiety score, there was a 1.7 and 1.8 point increase in the maltreatment score, respectively. These findings confirm the association of child maltreatment with depression and anxiety among DHH students.

## 4 Discussion

Parental abuse (including the use of physical or psychological punishment) and neglect undoubtedly have several short- and long-term, physical and psychological effects on the child (Gershoff and Grogan-Kaylor, 2016; Hammad and Awed, 2020; Kong et al., 2021a,b). Children who experience parental abuse often present with co-existing conditions such anxiety, depression, constant fear, lack of security and psychological stability, low self-esteem, and poor self-confidence and personal competence (Xing and Wang, 2013; Maxwell and Huprich, 2014; Taillieu et al., 2016; Kong et al., 2021b; Awed and Hammad, 2022; Liu et al., 2023). The main objective of the present study was to fill the gap in the literature on child maltreatment and its relationship with mental health outcomes in the DHH population, a minority group that is rarely researched upon, especially in Saudi Arabia. This study was the first of its kind (to the best of the researchers’ knowledge) to examine these issues in a sample of DHH students in Saudi Arabia by taking demographic variables into account. The results showed that more than 50% of the DHH students experienced at least a moderate level of maltreatment, and about 47.3% experienced severe to very severe levels. With a combined incidence of about 97%, this incidence of child abuse and neglect in the present sample is extremely concerning. Furthermore, while about 50% of the students reported having experienced sexual abuse, over 89% reported experiencing physical abuse, and nearly all students (99%) experienced emotional abuse and neglect. These findings corroborate those reported in several previous studies (e.g., Kvam, 2004; Fellinger et al., 2012; Schenkel et al., 2014; Wakeland et al., 2018; Admire and Ramirez, 2021), which reported high incidences of multiple types of abuse (physical, emotional, and sexual abuse, and neglect) among DHH individuals of different ages, including children.

Furthermore, significant group differences were observed in terms of their demographic characteristics, with men s, having parents with low education and income levels, and belonging to a family with 5 or more children exhibiting higher maltreatment scores. These findings offer additional insight into the socio-cultural factors that may be influencing the parent–child relationship with DHH children, and therefore, the latter’s vulnerability to abuse and neglect. As evidenced by a higher incidence of maltreatment in DHH children with hearing parents in the present study, communication and attitudes toward DHH children seem to play a vital role. Majority of DHH children estimated at over 95% by Hindley (2005) are born into hearing families with no previous experience of deafness (Landsberger et al., 2014). Hence, such parents often experience difficulties in communicating with their DHH child, and/or accommodating to their different needs (Opoku et al., 2022). This may lead to higher levels of parental frustration, and possibly a higher likelihood of using harsh disciplinary strategies (including but not limited to scolding, harsh language, and corporal punishment). Evidently, this may increase the DHH child’s vulnerability both physical and emotional abuse (Knutson et al., 2004). This is supported by the present results that DHH participants were less likely to experience maltreatment when they had DHH parents as compared to their peers with hearing parents. In addition (Schenkel et al., 2014) reported that the severity of hearing impairments was associated with lower levels of communication with others, which may put children at greater risk of abuse and aggression. Further, they added that DHH children are less likely to report or even talk about aggression with others, and therefore, perpetrators may feel reassured that they are not held accountable for the child abuse (Schenkel et al., 2014). Relatedly, hearing disability is associated with limited access to information through various media, or access to support from social institutions, thus mitigating their access to education, health services, or psychological support to reduce their vulnerability to abuse (Schenkel et al., 2014).

The present results also indicated a very high incidence of emotional abuse and neglect. This result needs to be examined in the context of cultural norms and values prevailing in some societies. For example, in the Lebanese society, which is similar to the Saudi society in terms of socio-cultural norms, it is considered normal for children to take care of themselves without parental supervision at a younger age (Sawrikar, 2014). With DHH children being dependent on the parents, siblings, or other close family members for a longer period, their dependence may be a source of stigma, isolation, and possible aggressive behavior toward them. Socio-cultural differences in what is acceptable parenting behavior is also an important factor. For instance, in several traditionalistic societies, parents’ or adults are considered as an authority figure and the use of some amounts of violence (e.g., smacking, scolding, corporal punishment) could be considered harmless and may even be viewed as appropriate parental supervision (Fekih-Romdhane et al., 2022). Especially in the case of DHH children in the Saudi society, keeping them away from certain experiences, controlling every aspect of their life, enforcing parental decisions on the child, etc., are considered a matter of caution or protective behavior toward the child and not as deprivation or emotional abuse.

With reference to gender, the present study found that men students exhibited higher maltreatment scores as compared to their women counterparts. This finding corroborated the higher incidence of child abuse and neglect among men s as compared to women s reported in previous studies (Almuneef M. A. et al., 2016; Fekih-Romdhane et al., 2022). This may partly be because women s are generally better at perceiving verbal and non-verbal behaviors. As explained by Darling and Heckert (2010), women s tend to be more polite and friendly as compared to men s, while men s appear more dominant and aggressive. Possibly, women children are better at observing and understanding parental behaviors, and thus modify their own behaviors to avoid punishment. Furthermore, in the Saudi society, men children bear responsibility to safeguard and continue the family name or pride. Therefore, they may be subject to stricter discipline as compared to women children.

The results also indicated that DHH students with parents with lower levels of education and income had higher scores on maltreatment. Parents with low education may also be likely to have low-paying jobs, and the related stressors from both these factors may strain their relationship with family members, including their children. Additionally, providing for a child with special needs, such as DHH children, may be an additional source of financial stress for them. Indeed, family income, specifically poverty at the individual and neighborhood levels, have been found to be closely related to a higher risk for experiencing child abuse and neglect (Pelton, 2015; Maguire-Jack and Font, 2017). Furthermore, having a large family to take care of could be another stressor (Mikolajczak et al., 2018). As found in the present study, DHH children belonging to families with over 5 children had higher maltreatment scores. This may be related to the financial and emotional stress on parents to meet all children’s needs (Lundberg et al., 1994; Mikolajczak et al., 2018). Another aspect of parents’ low educational level could be their lack of awareness of their child’s rights and confidence in their own parenting skills (Yaghoubi-Doust, 2013) reported that parents with higher education have higher self-confidence and awareness of their children’s rights, due to which they may refrain from using harsh parenting. Further, with limited knowledge, awareness, and education, such parents may be less adept at accessing and utilizing the social supports available to them as low-income families or as those taking care of a child with a disability. With limited social resources to tap into, these parents may rarely receive reprieve from their struggles of caring for a DHH child. This could also circle back to lack of resources to improve their skills in taking care of a DHH child, e.g., learning sign language and more effective communication strategies.

With reference to mental health outcomes, the current results also showed high incidences of depression and anxiety in the present sample, which could be partly attributed to their hearing status itself, and the struggles, stigma, and isolation that come with it, and partly to their experience of maltreatment. Previous studies have confirmed the association between child maltreatment and poor mental health among DHH individuals (Wright et al., 2009; Schenkel et al., 2014). It is well-established that repeated and prolonged maltreatment leads to feelings of worthlessness, shame, guilt, depression, anxiety, and low self-esteem among victim (Huang et al., 2010; Fakhari et al., 2012; Fellinger et al., 2012). Higher exposure to maltreatment is also linked to a higher degree of maladaptation and psychological incompatibility, thus suggesting that child maltreatment victims are also highly likely to exhibit behavioral and emotional dysfunction (Hampton, 1999). Further, as mentioned earlier, poor communication between parents and the related isolation and loneliness experienced by the DHH children may exacerbate their mental ill-health (Fan et al., 2023).

Though this study has several valuable findings, it is important to consider its limitations while drawing conclusions and practice implications.

### 4.1 Limitations of the study

The first set of limitations pertains to the sample utilized in the present study, which limit the generalizability of its findings. For instance, the sample only included DHH secondary students attending an integration program in schools in southern Saudi Arabia. Even considering the population of DHH children and adolescents, the limitations in terms of representation of age groups, geographic location and related socio-cultural differences, etc. cannot be ignored. Therefore, the present prevalence data, as well as other findings are more specific to the population that may share characteristics with the present limited sample of DHH secondary students attending an integration program in schools in southern Saudi Arabia. In reality, auditory and cultural definitions of the term “deaf” affect identity, group affiliation, and social concepts. In addition, the current sample included only DHH students from regular schools offering integration programs. Hence, DHH sample of DHH students may have unique sociocultural characteristics compared to DHH students who may not be similarly educated. As such, current results should have been interpreted considering the social and cultural characteristics of integrated DHH students. With integration, it is also important to note that the aspects of belongingness to the “Deaf” community and/or “hearing” community, and the individuals’, families, society’s perception of their “Deafness” were not included in the present study. This sense of belongingness with the “Deaf” community could act as a protective factor against maltreatment, long-term trauma, and mental health outcomes (Schenkel et al., 214). Further, it could be also linked to these children’s lack of identification with their hearing parents, which may be another factor causing frequent interpersonal problems among them. This too could explain the higher incidence of maltreatment among DHH students with hearing parents. Furthermore, there too, the present sample did not clarify if one or both parents’ hearing status did not match that of the participant. Having at least one parent with hearing impairments may change the dynamic in the household. However, unfortunately, the present dataset did not contain such information. Further, both maltreatment and mental health outcomes, and their interplay with hearing status could be influenced by several other socio-demographic factors not included in the present study. For instance, having another DHH sibling could be a protective factor against maltreatment (Schenkel et al., 2014). Additionally, parents’ mental health, their own experience of maltreatment as children or adults, personality factors, etc., could influence their relationship with their children, including the DHH child. In future, similar studies on DHH children and adolescents should aim to include different socio-cultural factors.

Finally, as with all cross-sectional studies, the causal relationship between child maltreatment and mental health outcomes cannot be established. The participants’ depression and anxiety could precede their experience of maltreatment, and could be factors that rendered them vulnerable to maltreatment owning to the cognitive, perceptual, and emotional effects of these mental illnesses on the individual. Further, the maltreatment and mental health outcomes could have a bidirectional relationship augmenting each other’s effects. Future longitudinal, controlled, and/or qualitative studies could shed more light on these relationships.

Despite these limitations, current findings possess merit as they add to the extant literature by exploring a relatively unresearched topic on the present target population, especially in Saudi Arabia. This preliminary study opens up prospects for future research in this area. Further examination of the impact of parental abuse on different aspects of DHH students’ development, such as psychological, emotional, social, cognitive, and academic aspects, may provide valuable information that can be used to develop effective interventions and training programs. Finally, more research may be needed on the prevalence and impact of peer or teacher abuse and bullying on DHH children. With these strengths, the present study offers important insights and practice implications.

## 5 Conclusions and implications

The main objective of this study was to determine the prevalence and mental-health effects of child maltreatment on the present sample of DHH students in Saudi Arabia. Our findings showed that a large majority of the participants experienced moderate to very severe levels of various types of abuse and neglect, and all of them experienced at least one type to at least some extent. Further, statistically significant differences based on gender, parents’ educational and income level, number of children in the family, and parents’ hearing status revealed important socio-demographic factors that may contribute to the DHH students vulnerability to abuse and neglect. Finally, the results of the linear regression analysis indicated that depression and anxiety predicted the prevalence of maltreatment, explaining over 80% of the variance in maltreatment scores. When combined with past research in this field, the present findings provide strong evidence of the negative impact of child abuse and neglect on the mental health of DHH students, and vice versa. Therefore, it is important to recognize parental abuse among DHH children and adolescents as a public health concern and to develop appropriate strategies to prevent the same. Provision of support to parents in terms of socio-economic stressors, parent–child communication, and the conscious or unconscious bias against DHH children would go a long way in preventing the triggers that may lead them to engage in abusive behaviors toward their DHH child. Further, different professional, and governmental and non-governmental agencies working with DHH individuals and their families, including those in the field of public health (physical and mental health), child protection, justice, law, etc., need to consider integrated services that cater to the needs of the DHH individuals and their family as a whole. In the present study, communication difficulties and the social isolation experienced by DHH children within their families, especially when they belong to families without other DHH individuals (e.g., parents or siblings), emerged as one of the risk factors for parental abuse. Therefore, focus should be on providing parents skills and knowledge related to effective communication skills (e.g., sign language), appropriate parenting strategies (e.g., conflict resolution and disciplining), and effectively utilizing the existing government aids and services, and their social network to tackle their parenting challenges. Finally, considering DHH individuals or those with any visible or invisible disability, reducing the social stigma associated with their disability is an important part of achieving long-term protection efforts. False stereotypes about and negative views of DHH individuals can lead to increased feelings of shame and inferiority in the individuals themselves, and in their immediate family members, leading to greater social isolation, anxiety, depression, and the risk of maltreatment. Therefore, awareness programs, diversity and inclusion measures, appropriate accommodations, etc., are required to improve the overall quality of life of DHH individuals and their family, and to reduce their vulnerability to maltreatment within and outside their family.

## Data availability statement

The original contributions presented in the study are included in the article/supplementary material, further inquiries can be directed to the corresponding author.

## Ethics statement

The studies involving humans were approved by the authors complied with APA ethical standards, and the ethics guidelines of Najran University. The study was approved by the Deanship of Scientific Research at Najran University (NU/IFC/2SEHRC/-/27). Participation in the research was voluntary and informed consent was obtained from all participants. The studies were conducted in accordance with the local legislation and institutional requirements. Voluntary informed consent was provided by the participants’ legal guardian/next of kin.

## Author contributions

MH: Conceptualization, Data curation, Methodology, Writing – original draft, Writing – review & editing. MA-O: Conceptualization, Methodology, Writing – original draft. HA: Conceptualization, Methodology, Writing – original draft.
